# Dealing with Corticosteroid and High-Dose Cyclosporine Therapy in a Pyoderma Gangrenosum Patient Contracting a COVID-19 Infection

**DOI:** 10.3390/jpm12020173

**Published:** 2022-01-27

**Authors:** Marcella Ricardis May, Albert Rübben, Andrea Lennertz, Luk Vanstreels, Marike Leijs

**Affiliations:** 1 Department of Dermatology and Allergology, University Hospital of the RWTH Aachen University, 52074 Aachen, Germany; marcella.may@rwth-aachen.de (M.R.M.); Albert.Ruebben@post.rwth-aachen.de (A.R.); 2 Department of Dermatology, St. Nikolaus Hospital, 4700 Eupen, Belgium; luk.vanstreels@hospital-eupen.be; 3 Department of Internal Medicine, St. Nikolaus Hospital, 4700 Eupen, Belgium; andrea.lennertz@hospital-eupen.be

**Keywords:** COVID-19, pyoderma gangrenosum, immunosuppression, cyclosporine, corticosteroids, autoinflammatory disease

## Abstract

Pyoderma gangrenosum (PG) is a rare and chronic neutrophil inflammation belonging to the spectrum of autoinflammatory disorders. Immunosuppressive therapy is the cornerstone of successful treatment. However, due to the global COVID-19 pandemic, physicians struggle with therapeutic strategies during infection. This paper describes the case of a 58-year-old patient with a very painful, rapidly increasing wound on his right foot, which was diagnosed as pyoderma gangrenosum. Five weeks after the initial treatment with high-dose immunosuppressives (combination therapy with cyclosporine A and systemic methylprednisolone), he became infected with COVID-19. Reduction in the immunosuppressive dosage proved effective, as the patient recovered from COVID-19 without any complication and showed rapid wound healing.

## 1. Introduction

Currently, we are facing a global pandemic with severe acute respiratory syndrome coronavirus 2 (SARS-CoV-2) infection. While vaccines diminished the number of new infections, hospital admissions, and deaths, the number of so-called breakthrough hospital admissions of fully vaccinated individuals is increasing. Vulnerable patients with immunosuppressive medication, in particular, are at high risk of suffering from severe symptoms during an infection. In addition, there is always a part of the population that is not vaccinated. This can be due to underlying medical conditions or because a few individuals refuse to be vaccinated. Studies reported that immunosuppressed and multimorbid patients have a poor immune response following the SARS-CoV-2 vaccination [1]. In addition, experiences with other coronaviruses showed that immunosuppression could lead to atypical presentations including prolonged incubation periods, a persistence in asymptomatic viral shedding, and atypical symptoms such as gastroenterological disease and encephalitis. Experience and research evidence on COVID-19 in such patients are still very limited [2,3,4,5,6,7]. The severity of the COVID-19 infection is correlated with age, as well as gender and comorbidities such as cardiovascular diseases, diabetes, chronic respiratory diseases, hypertension, and cancers. A measured high number of white blood cells and neutrophils, as well as elevated D-dimer levels, are observed in COVID-19 patients with severe outcomes [8,9]. 

Pyoderma gangrenosum (PG) is a rare and chronic neutrophil inflammation. It belongs to the spectrum of autoinflammatory diseases and is characterized by recurrent episodes of progressive and often painful sterile inflammation. PG can be found solitary and multiple. According to Frank C. Powell (1996), six forms can be classified [10]. The classic and most frequent form is the ulcerous form. It develops fast and is mostly laminar. PG is a rare disease, as the global incidence is estimated to be around three to ten cases per one million population per year [9]. Moreover, it is frequently associated with several comorbidities especially inflammatory intestinal diseases (IBDs) (25%) [11], autoimmune disease (14.1%), hematological disorder (6.2%), and others (17.2%) [9]. PG is more often present in women than in men and most prevalent in 50–70-year-old individuals. Diagnosing PG properly is indispensable since PG is linked with a major reduction in quality of life and even with death [8]. To avoid misdiagnosis the Delphi score (D), as well as the so-called PARACELSUS score (P), which is an acronym, considers the following criteria (Table 1) [12,13]:

The pathogenesis of this autoinflammatory disease is not just multifactorial but also still unknown. In addition, the different PG types are differing in pathogenesis according to their form. Abnormalities in the function of the immune system including inflammatory cytokines, neutrophilic dysfunction, and specific genetic mutations have been observed. Evaluation of 21 patients with PG demonstrated infiltrates of CD-31 T cells and CD-1631 macrophages with high levels of interleukin (IL)-8 in the lesion. As IL-8 is a potent chemotactic for neutrophils, this explains the typical neutrophilic infiltrates, which can be visible in histopathological examination [14]. Overexpression of interleukin (IL)-1β plays an important role in PG as well but is not specific [15]. It has been suggested that PG and rheumatic diseases share a commonality in T-cell abnormalities. It was proposed that autoreactive T cells, which destroy pilosebaceous units, contribute to the pathophysiology since complete hair loss is found after healing [16]. An elevation of several chemotactic cytokines and interleukins was found in another study: CXC motif chemokine ligand (CXCL) 9, CXCL 10, CXCL 11, chemokine C-C ligand 3 (CCL-3) and CCL-5, interleukin (IL)-36G, IL-17A, IL-8 (a neutrophil chemokine), and interferon gamma have been identified in PG lesions. In addition, an upregulation of certain transcription factors (STAT 1 and STAT 4) responsible for promoting Th1 differentiation was found in another study [17]. Very rare PG can appear as a monogenetic disease such as the PAPA syndrome in which case it is accompanied by arthritis and cystic acne [18]. Early diagnosis of pyoderma gangrenosum is important in order to minimize the size and necrotic painful character of the ulcer, as well as the formation of disfiguring scars [16]. In addition, secondary bacterial infections can occur and should be prevented. 

Immunocompromised patients, including those requiring immunosuppressive therapy following autoinflammatory skin disease, are at high risk for developing severe disease following SARS-CoV-2 infection [2]. Neither the data regarding the best medical management of immune-compromised patients testing positive for SARS-CoV-2 nor strategies for reducing or modifying immunosuppression are easy to standardize yet [2]. In this case, in this study, we describe a 58-year-old man who became infected with COVID-19 while being treated with (high-dose) corticosteroids and cyclosporine. As cases similar to this are not commonly discussed, the aim of this paper was to set an example for other clinicians of how an adjustment of immunosuppressive therapy in patients with a rare autoinflammatory disease such as PG contracting a COVID-19 infection could be successfully performed.

## 2. Case Report

**Figure 1:**
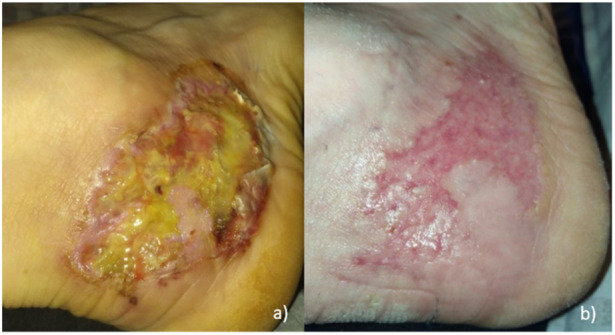
Pictures of the ulcer: (**a**) at the time of hospitalization; (**b**) healed ulcer 8.5 weeks after initial diagnosis.

We present a 58-year-old Caucasian European male with an extremely painful ulcer at his right lateral malleolus (see Figure 1a). Anamnesis revealed that the wound appeared after massage therapy that he received for Achilles tendinitis. Initially, he reported a painful bulla, which became ulcerative. Due to other physical problems (otitis media and necrosis of the temporomandibular joint) and the fact that he was taking antibiotics and analgesics, the patient did not seek medical treatment at an earlier stage of the disease. However, the wound expanded within two weeks rapidly and became very painful. Medical history revealed comorbidities of diverticulosis and hypercholesterolemia.

Clinical evaluation revealed a 4 cm × 8 cm ulcer with tissue necrosis, as well as a very painful livid erythematous encircling undermined edge (Figure 1a). No signs of venous insufficiency were visible. Clinical assessment showed normal peripheral vascularization.

An infectious ulcer was excluded by a negative microbiological swab. Peripheral arterial occlusive disease and venous insufficiency could be excluded with duplex sonography. Computed tomography of the foot and ankle was used to exclude osteomyelitis. 

Two skin biopsies were taken from the edge of the ulcus. As seen more often in PG, histopathological findings were not specific. However, the dermis showed fibrosis including mixed cellular inflammatory infiltrates. These were mainly perivascular lymphocytic but also mixed lymphohistiocytic infiltrates including some neutrophil granulocytes. Infiltrates contained mostly CD-3 positive T lymphocytes. 

We diagnosed the ulcer as an ulcerating form of pyoderma gangrenosum due to its typical clinical presentation and as the minor criteria (Delphi) and major and minor criteria of the PARACELSUS score of pyoderma gangrenosum (which is discussed in detail in the Introduction Section) were fulfilled [12]. 

We decided to start directly a combination therapy with (2.5 mg/kg) cyclosporine A (CsA) twice a day and systemic methylprednisolone (1 mg/kg) per day because of severe pain and a rapidly increasing wound (despite premedication with 20 mg methylprednisolone from the patients’ otorhinolaryngologist) and in order to avoid side effects of long-term steroid usage [8]. A local corticosteroid and a calcineurin inhibitor ointment were applied once a day, and an adsorbing dressing was added. 

An improvement in pain and a decrease in ulcer size were observed. Moreover, the C-reactive protein (CRP) declined satisfactorily under this treatment. After 5 days of treatment, the CRP declined from 198.3 mg/L (see Table 2) to 37.7 mg/L (RV < 8.0).

**Figure 2:**
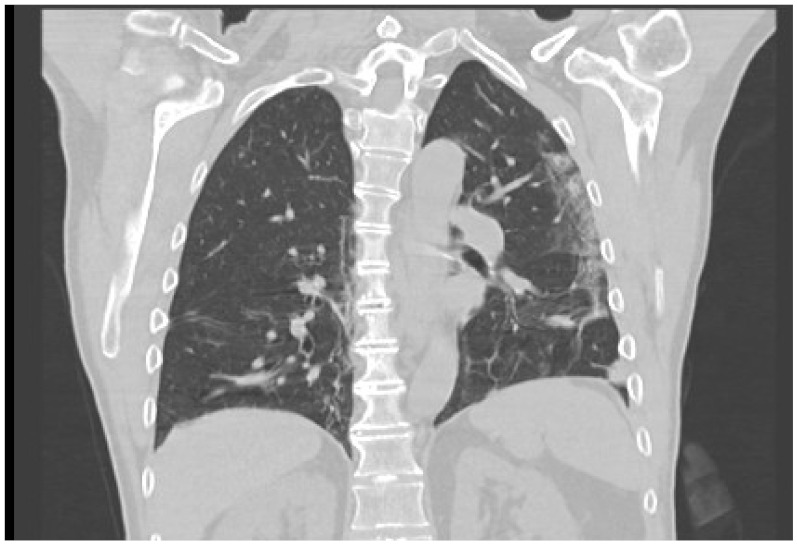
Typical COVID-19 lung infiltrates, five weeks after initial presentation.

**Figure 3:**
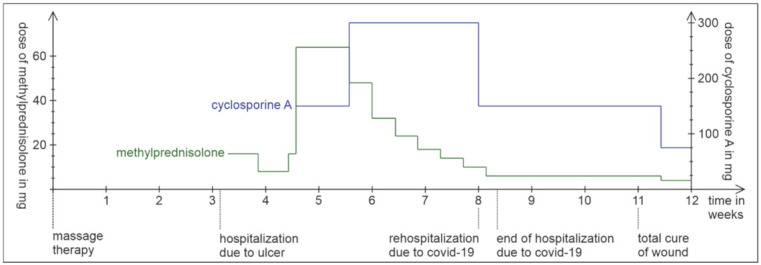
Timeline of patient’s healing and the dose of methylprednisolone and cyclosporine A.

Five weeks after the initial hospitalization, the patient presented to the emergency care with fever and dyspnea. At this time, he was still taking 300 mg CsA and 8 mg methylprednisolone. A PCR test (long nasal swab) confirmed the SARS-CoV-2 infection. Computer tomography demonstrated typical lung infiltration (Figure 2). It had to be decided whether to continue or modify the immunosuppressive therapy or to interrupt cyclosporine A since cyclosporine interferes with the antiviral immune pathway [3]. We decided to halve the cyclosporine A dosage to 75 mg twice a day (Figure 3). The patient’s clinical condition significantly improved within three days from the beginning of COVID-19 symptoms. The ulcer healed 8.5 weeks after the initial diagnosis (Figure 1b). 

## 3. Discussion

Immunosuppressive therapy—mostly corticosteroids in combination with CsA—depending on the severity, the extent of the PG lesion, and previous conditions, is the cornerstone of the treatment in PG patients. In addition, proper local treatment, including adapted wound care to prevent secondary bacterial infection and slight compression, as well as administration of analgesics for pain relief, is important [16]. In the case of our patient, pain relief and reduction in wound size occurred after initiating the therapy with corticosteroids and CsA. At the time of treatment, the patient was not vaccinated yet, and as the patient contracted a COVID-19 infection, we needed to decide how to continue the immunosuppressive therapy.

Patients with severe COVID-19 infections may receive systemic corticosteroids in order to reduce the systemic inflammatory response that can lead to lung injury and multisystem organ dysfunction [4]. The uncontrolled proinflammatory response with the release of cytokines known as the cytokine storm is not well defined. It has been reported to occur after unsuccessful viral clearance and it might involve less specific defense mechanisms such as monocyte and macrophage activation [19]. The exact pathophysiologic mechanisms are unclear. One study reported elevated IL-6 levels in severely ill COVID-19 patients [20]. Conversely, another study on 46 critically ill COVID-19 patients showed no difference in levels of IL-6/8 and tumor necrosis factor (TNF), compared with other critically ill patients, and even lower levels than in critically ill patients with a septic shock [21]. 

How to deal with immune suppressive medication during COVID-19 infection is not standardized yet. One study on 374 clinician-reported psoriasis patients from 25 countries showed that the hospitalization rate due to COVID-19 was more frequent in patients using nonbiologic systemic therapy than in those using biologics (OR = 2.84; 95% CI = 1.31–6.18). Other risk factors in this study were older age, male sex, nonwhite ethnicity, and comorbid chronic lung disease [22]. Some studies advise withholding immune-modulating medication, with the exception of corticosteroids [5]. Contrarily, a recent position statement on immunotherapeutics in patients with inflammatory skin disease pointed out that limited data suggested there is no additional risk for patients using CsA. However, no dose-dependent advice was given in this paper [23]. CsA has already been used as a treatment for patients with COVID-19, as reported in the World Health Organization (WHO) guidelines [4,24,25]. CsA bind to cyclophilin, which theoretically inhibits SARS-CoV-2 replication [26]. In agreement, in another review, it was concluded as well that “there is no evidence that [the] use of cyclosporine possesses an additional risk for severe COVID-19” [27]. Other studies on immunosuppressives during COVID-19 infection suggest that cyclosporine A can potentially prevent acute respiratory failure and hyperinflammation-induced lung injury [28,29]. On the other hand, an increased risk of several viral infections is seen in organ transplant patients treated with CsA [30]. As a causative factor, CsA suppresses helping T cells and precursors of cytotoxic T lymphocytes and depresses the innate immune system by inhibition of natural killer cells, which are the most important immune cells in this regard, along with antibody-dependent cellular cytotoxicity and certain cytokines (IL-6, IL-2), prominently interferons. NF-κB suppression inhibits mainly the production of proinflammatory cytokines. An animal study demonstrated an inability to mount an effective immune response to viral infections with the administration of CsA [31]. Even though a potential beneficial effect of cyclosporine has been discussed in several studies, the experts are not yet unified regarding this topic. In addition, most studies do not give dose-dependent advice for CsA. When administrating CsA we have to keep in mind that it will inhibit establishing immune memory following COVID-19 infection or vaccination [32].

**Figure 4:**
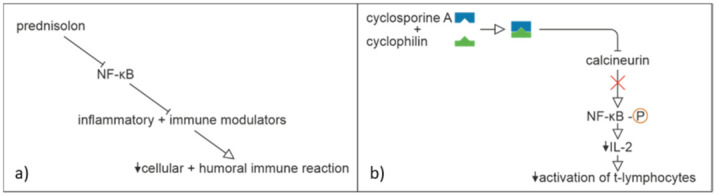
Effect of (**a**) prednisolone and (**b**) cyclosporine A.

In the presented case, methylprednisolone and cyclosporine were the main therapy of the patient. Their specific immunosuppressive mechanisms are described in Figure 2. Methylprednisolone is a synthetic glucocorticoid and displays the same features (immunosuppressive, anti-inflammatory, and antiallergic) as cortisol. Corticosteroids inhibit both T and B cells. It rapidly reduces circulating T cells due to enhanced circulatory emigration, induction of apoptosis, inhibition of T-cell growth factors, and impaired release of cells from lymphoid tissues. Effects on B-cell function and immunoglobulin production are more delayed [33]. Potent suppressive effects on the effector functions of monocytes and neutrophils, as well as the suppression of NF-κB and activator protein- 1 (AP-1), are of great importance because they inhibit many inflammatory and immune modulators as a result of modulation of the expression of pro-inflammatory cytokines such as IL-1b, TNFα, and IL-2 (Figure 4) [34]. As a result, the cellular and humoral immune reactions regress. In addition, suppression of NF-κB can suppress the synthesis of cyclooxygenase-2 (COX-2). In conclusion, the glucocorticoid responsive genes encode a large array of effectors in the host antiviral defense system [35]. Synergistically, cyclosporine A binds to cyclophilin, which leads to an inhibition of calcineurin, leading additionally to inhibition of intracellular NF-κB (Figure 4) [3]. As a consequence, the transcription factor NF-AT cannot be dephosphorylated, and the cytokine IL-2 is produced in lower quantities. Consequently, T lymphocytes are activated less. Interestingly, a mutation in the NF-κB signaling pathway (critically important for regulation of the innate and adaptive immune responses) has been identified in a PG case report [36]. It has been postulated that observed effects of corticosteroids are dose dependent probably due to the variable dose-dependent affinity of target genomic sites for the glucocorticoid receptor and that some additional genes are affected as the concentration of glucocorticoid increases [34]. It has been proven that corticosteroids improve survival of critically ill COVID-19 patients; nevertheless, a recent study showed that a dose over 40 mg methylprednisolone equivalent dosing (MED) had a higher mortality rate [37]. In the aforementioned recent position statement, it was advised to continue corticosteroids at a lower dose < 10 mg prednisolone since it may suppress viral clearance [30]. 

Alternative treatment of pyoderma gangrenosum includes azathioprine, methotrexate, thalidomide, dapsone, mycophenolate mofetil, sulfasalazine, cyclophosphamides, colchicine, antitumor necrosis factor-alpha inhibitor (TNF-α), and intravenous immune globulin (IVIG). As our patient reacted well to the combinational therapy with cyclosporine and corticosteroids, alternative therapy was not taken into consideration. Additional therapeutic options include antibodies against IL-17, IL-12/IL-23, Il-6, IL-1β, IL-1 receptor I, as well as JAK1, 2, and 3 inhibitors [16,38,39,40,41,42,43,44]. For all the mentioned therapeutic options, and likewise for CsA, limited data demonstrated no risk for severe COVID-19 infection [30]. 

In conclusion, we cautiously advise the adaption of immune suppressive therapy in patients with pyoderma gangrenosum suffering from COVID-19. Adaptation should be performed according to the severity of the disease and the stage of treatment. In patients with symptomatic light to moderate infection, we suggest temporarily halving the CsA treatment in case of high dosage and lowering the corticosteroid dose. 

## Figures and Tables

**Table 1:** The Major and Minor Criteria of the Delphi and PARACELSUS score.

Major Criteria	Minor Criteria
Rapidly progressing course of disease (P)	Prompt alleviation of symptoms by immunosuppressants (P *)
Reddish-violaceous wound border (P)	Characteristically irregular shape of the ulcer (P)
Exclusion of relevant differential diagnoses (P)	Extreme pain (P)
Neutrophilic infiltrate found in the ulcer edge (D)	Lesion at site of trauma (P)
	Exclusion of infection (D)
	Pathergy (D)
	History of inflammatory bowel disease or inflammatory arthitis (D)
	History of papule, pustule, or vesicle ulcerating within 4 days of appearing (D)
	Peripheral erythema, undermining border, and tenderness at ulceration site; (D)
	multiple ulcerations, at least 1 on an anterior lower leg (D)
	Cribriform or “wrinkled paper” scar(s) at healed ulcer sites (D)
	Decreased ulcer size within 1 month of initiating immunosuppressive medication(s) (D)

* The Paracelsus score contains three additional criteria: suppurative inflammation in histopathology, undermined wound border, systemic disease associated.

**Table 2:** The laboratory parameters at the time of the patient’s hospitalization.

Laboratory Parameter	Value	Reference
Hemoglobin	9.8 g/dL	13.3–17.7 g/dL
Hematocrite	30%	40–52%
Red Blood Cells	3.67 × 10^6^/mm^3^	4.40–5.90 × 10^6^/mm^3^
Blood Platelets (in Ethylenediaminetetraacetic Acid (EDTA))	544 million/μL	3.9–10.6 million/μL
Reticulocytes	2.05%	0.50–2.00%
Neutrophiles	70.7%	40.0–70.0%
Lymphocytes	14.3%	20.0–45.0%
Eosinohiles	0.7%	<4.0%
Basophiles	0.4%	<2.0%
Monocytes	13.9%	<12.0%
Creatinin	0.55 mg/dL	0.80–1.30 mg/dL
Uric Acid	4.5 mg/dL	2.6–7.2 mg/dL
Sodium	133 mmol/L	135–145 mmol/L
Potassium	4.6 mmol/L	3.5–5.1 mmol/L
Chloride	99 mmol/L	97–107 mmol/L
**CRP (c-reactive proteine ultrasensitive)**	**198.3 mg/L**	**<8.0 mg/L**
Protein total	60.3 g/L	58.0–83.0 g/L
AST (spartate aminotransferase)	31 U/L	<42 U/L
GPT (alanine transaminase)	33 U/L	<40 U/L
LDH (lactaatdehydrogenase)	125 U/L	<250 U/L
Alkaline Phosphatases	57 U/L	30–120 U/L
Gamma-GT (glutamyl transferase)	34 U/L	<50 U/L
Bilirubin Total	0.4 mg/dL	0.2–1.2 mg/dL
Bilirubin Direct	0.1 mg/dL	<0.3 mg/dL

## Data Availability

Most data are presented in the manuscript. Further details are available on request from the corresponding author. The data are not publicly available due to privacy restrictions.
